# Fibroblast growth factor 23: Regulation, signalling and systemic links between bone, metabolism and inflammation

**DOI:** 10.1016/j.molmet.2026.102353

**Published:** 2026-03-14

**Authors:** Antía Crespo-Golmar, María Piñeiro-Ramil, Andrés Pazos-Pérez, María Crugeiras-Sampedro, Lorena Zas-Veiga, Djedjiga Ait Eldjoudi, Ana Suárez-Rodríguez, Alberto Jorge-Mora, Ana Alonso-Pérez, Rodolfo Gómez

**Affiliations:** Musculoskeletal Pathology Group, Health Research Institute of Santiago de Compostela (IDIS), Santiago University Clinical Hospital, SERGAS, 15706 Santiago de Compostela, Spain

**Keywords:** Phosphatonin, Mineralisation, Mineral metabolism, Chronic kidney disease, Cardiovascular disease

## Abstract

Fibroblast growth factor 23 (FGF23) is a bone-derived hormone and growth factor that plays a central role in phosphate and mineral homeostasis. Beyond its classical endocrine actions on the kidney, accumulating evidence indicates that FGF23 exerts broad paracrine and systemic effects that extend to metabolism, inflammation, cardiovascular function and cancer biology. FGF23 production is tightly regulated at the transcriptional and post-translational levels in osteocytes and osteoblasts, resulting in the release of either intact bioactive FGF23 or cleaved fragments with distinct biological properties. Canonical FGF23 signalling requires fibroblast growth factor receptors in complex with the co-receptor α-Klotho, although α-Klotho-independent pathways have been described in several tissues. This review provides a comprehensive overview of FGF23 biology, focusing on its regulation, processing and signalling mechanisms, and integrating current knowledge of its paracrine and endocrine actions across multiple organ systems. We discuss the role of FGF23 in bone mineralisation, phosphocalcium metabolism and energy homeostasis, as well as its involvement in inflammatory states, anaemia, cardiovascular disease, chronic kidney disease, cancer and nervous system function. Experimental and clinical evidence supporting both adaptive and maladaptive roles of FGF23 in health and disease is critically examined. Overall, FGF23 emerges as a multifunctional growth factor and hormone that links bone to systemic metabolic and inflammatory networks. Understanding the context-dependent actions of FGF23 may provide novel insights into disease mechanisms and identify new opportunities for therapeutic intervention.

## Highlights

• FGF23 integrates bone-derived signalling with systemic metabolic regulation.
• Post-translational processing critically shapes FGF23 signalling outcomes.
• FGF23 exerts paracrine effects on bone mineralisation and osteocyte function.
• Endocrine FGF23 links mineral metabolism to inflammation and energy balance.
• Dysregulated FGF23 contributes to cardiovascular, renal and systemic disease.

## Graphical abstract

**Figure:**
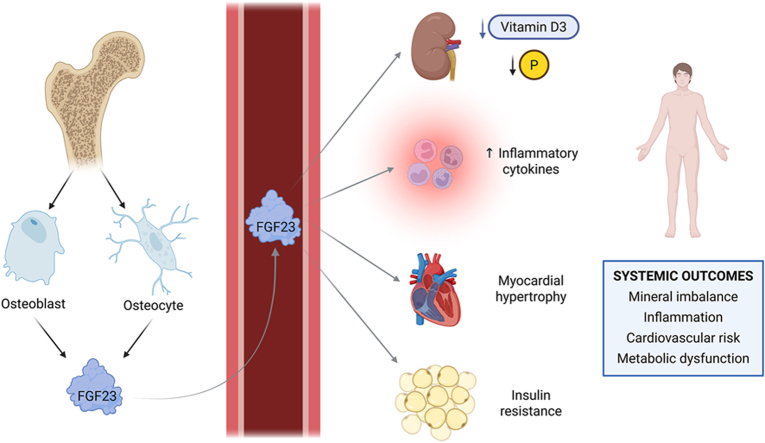

## 1 Introduction

Fibroblast growth factor 23 (FGF23) is a member of the fibroblast growth factors family that functions as both a hormone and a growth factor with broad systemic actions. Although FGF23 is predominantly synthesized and secreted by osteocytes and osteoblasts [[1], [2], [3], [4]], lower levels of expression have been reported in several non-skeletal tissues such as kidney, liver, heart, and brain [[5], [6], [7]]. Initially characterized as a key regulator of phosphate homeostasis and mineral metabolism, FGF23 is now recognized as a multifunctional signalling molecule with broader effects, including implications in cardiovascular and renal pathologies, haematopoiesis, and systemic inflammation [[8], [9], [10]].

## 2 Structure and signalling of FGF23

### 2.1 Post-translational processing of FGF23

**Figure 1:**
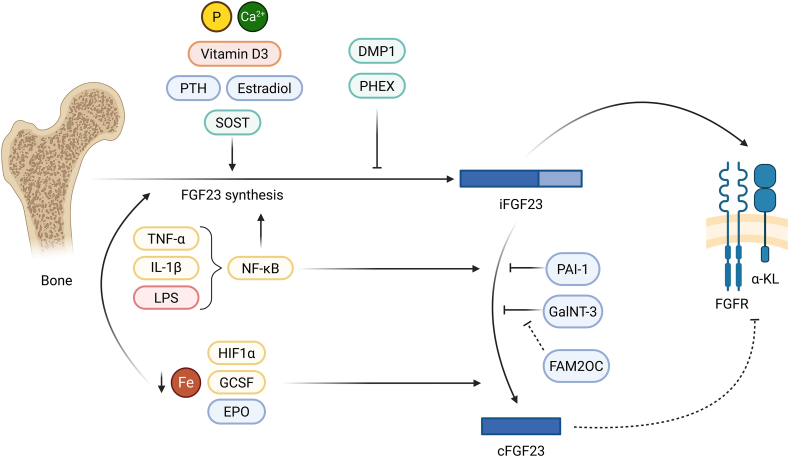
**Regulation and signalling of FGF23.** FGF23 is primarily produced by osteocytes and osteoblasts and released as intact bioactive FGF23 (iFGF23). Its synthesis is stimulated by mineral and hormonal factors, including phosphate, calcium, active vitamin D3, parathyroid hormone (PTH), 17β-estradiol and sclerostin (SOST), and is negatively regulated at the local bone level by dentin matrix acidic phosphoprotein 1 (DMP1) and phosphate regulating endopeptidase homolog X-linked (PHEX). Post-translational processing of iFGF23 results in its cleavage into C-terminal fragments (cFGF23), a process inhibited by plasminogen activator inhibitor-1 (PAI-1) and O-glycosylation mediated by N-acetylgalactosaminyltransferase 3 (GalNT3). Phosphorylation by FAM20C reduces GalNT3-dependent glycosylation, thereby favouring FGF23 cleavage. Inflammatory stimuli, including tumour necrosis factor (TNF), interleukin-1β (IL-1β) and lipopolysaccharide (LPS), induce FGF23 transcription and cleavage via NF-κB signalling. Iron deficiency and hypoxia-related pathways, involving hypoxia-inducible factor-1α (HIF-1α), granulocyte colony-stimulating factor (G-CSF) and erythropoietin (EPO), also promote increased FGF23 synthesis and processing. Intact FGF23 signals through fibroblast growth factor receptors (FGFRs) in complex with the co-receptor α-Klotho (α-KL), whereas cFGF23 is proposed to antagonise this signalling by competing for receptor binding.

The biological actions of FGF23 are determined by its post-translational processing and receptor-dependent signalling mechanisms. The FGF23 gene is composed of three exons and encodes a protein of 251 amino acids (aa). The N-terminal region, a 24-aa signal sequence, is followed by a core which constitutes an FGF homology domain. The C-terminus region of FGF23 is essential to its biological activity since it is the binding site to its receptor, the α-Klotho (α-KL)/Fibroblast growth factor receptor (FGFR) complex [[11], [12], [13]]. Once synthesized, two forms of mature FGF23 can be released into the systemic circulation: a 32.5 kDa bioactive intact protein (iFGF23) and a 10 kDa cleaved fragment (cFGF23), which is formed after iFGF23 undergoes intracellular proteolysis by subtilisin-like proprotein convertases [[14], [15], [16]]. It is thought that cFGF23 competes with iFGF23 for binding with the α-KL/FGFR complex, establishing a downregulation mechanism of the pathway [11]. The transformation of iFGF23 into cFGF23 can be prevented by plasminogen activator inhibitor-1 (PAI-1) [15] and also by N-acetylgalactosaminyltransferase 3 (GalNT3), which catalyses the O-glycosylation of FGF23 at Thr178 [17]. Conversely, phosphorylation at Ser180 by the protein kinase family with sequence similarity-20 member C (FAM20C) reduces O-glycosylation, and thus favours the production of cFGF23 [14,18,19] (Figure 1). The circumstances which determine the balance between bioactive and cleaved FGF23 remain uncertain.

### 2.2 α-Klotho-dependent and -independent signalling

The canonical actions of FGF23 are mediated through the complex formed by FGF23, FGFR (R1c, R3c, or R4), and α-KL [[20], [21], [22]]. These include mineral homeostasis (phosphate, calcium, and sodium), active vitamin D3 (1,25-dihydroxy vitamin D3) regulation, modulation of angiotensin-converting enzyme 2 (ACE2) synthesis, and α-KL production [[23], [24], [25]]. Nonetheless, FGF23 signalling in the absence of α-KL has been described in some circumstances, such as the pathogenesis of left ventricular hypertrophy [26], bone mineralisation [25] and hepatic production of inflammatory cytokines in chronic kidney disease (CKD) [27].

FGFR is an ubiquitous tyrosine kinase receptor with three immunoglobulin-like domains [28], while α-KL is a transmembrane protein mainly expressed in renal tubules, arteries, and the nervous and endocrine systems [29,30], although it has also been observed in mesenchymal tissues from bone marrow and growth plates [31,32]. α-KL has a small cytoplasmic domain of 11 aa and an extracellular domain of 980 aa formed by two homologous regions, KL1 and KL2, which bind to FGF23 [[28], [29], [30]]. Furthermore, transmembrane α-KL (α-KL™) can undergo cleavage of its extracellular domain in the distal renal tubule by ADAM proteases, producing a soluble α-KL ectodomain (α-KL^ecto^) that can be released into the systemic circulation and act in tissues in which α-KL™ is not expressed [28]. Both α-KL™ and α-KL^ecto^ exert FGF23-dependent functions as part of the FGFR/α-KL complex, thus increasing the ability of FGF23 to activate FGFR [20,28]. In addition, there is another soluble KL (sKL) isoform generated by alternative splicing that contains part of the extracellular domain, including KL1, with unknown function [29]. Collectively, the complex regulation of FGF23 post-translational processing and the diversity of FGFR and α-Klotho interactions define the range of signalling outputs of this growth factor.

## 3 Modulation of FGF23 expression

### 3.1 Systemic regulators of FGF23

The expression of FGF23 is regulated by a wide range of metabolic, inflammatory and hormonal stimuli. Several molecules involved in mineral metabolism increase the synthesis of this phosphatonin, including serum phosphate [21,[33], [34], [35], [36]], calciprotein particles [37], active vitamin D3 [[38], [39], [40], [41], [42], [43]], Notch [1], parathyroid hormone (PTH) [[44], [45], [46], [47], [48]] through Nuclear receptor-related 1 protein (Nurr1) [49], sclerostin [50], and 17β-estradiol [36]. In the field of inflammation, an increase in the transcription of FGF23 dependent on Nuclear Factor Kappa B (NFκB) can be induced by tumour necrosis factor (TNF) [51], interleukin 1 beta (IL-1β) [[51], [52], [53]], bacterial lipopolysaccharide (LPS) [51,52,54,55], lactic acid [56], oxidative stress-induced apoptosis [57], p38 mitogen-activated protein kinase (p38MAPK) [58], and serine/threonine protein kinase C (PKC) [59]. This is related to an increase in cFGF23 while maintaining a stable or slightly elevated iFGF23 [51,52]. FGF23 transcription and subsequent cleavage are also increased by iron deficiency [[60], [61], [62]], hypoxia-inducible factor 1 alpha subunit (HIF1α) [52,60,63], granulocyte colony-stimulating factor (G-CSF) [64], and erythropoietin (EPO) [9,63,65]. Oncostatin M, a mediator of myocardial remodelling, also increases FGF23 in the setting of heart failure [66].

Regarding diet, an increase in serum FGF23 has been associated with high intake of phosphorus [33,34], calcium [67,68], and animal protein [68], and with low levels of iron [60], magnesium, vegetable protein, and fibre [68]. Leptin [69,70], homocysteine [71], low-magnitude high-frequency vibration [72], mechanical tension [73], and over strenuous exercise [74] have also been associated with elevated FGF23. An increase in the expression of FGF23 was observed in children with a previous history of allogeneic solid organ transplantation who were receiving immunosuppressive treatment with triple therapy (calcineurin inhibitor, methylprednisolone, and an antimetabolite) [75]. FGF23 overexpression was also induced after an intravenous administration of saccharated ferric oxide during treatment of iron-deficiency anaemia [76]. Cadmium intoxication has also been described to suppress proteolytic processing of FGF23 and increase its serum concentration [77].

### 3.2 Bone-intrinsic regulation of FGF23

At the local bone level, two key regulators act as inhibitors of FGF23 transcription: Dentin Matrix Acidic Phosphoprotein 1 (DMP1) and Phosphate Regulating Endopeptidase Homolog X-Linked (PHEX). Pathogenic variants in these genes are associated with autosomal recessive and X-linked hypophosphatemic rickets, respectively, due to increased FGF23 [[78], [79], [80], [81], [82], [83], [84], [85], [86]]. A decrease in FGF23 has been associated with a glucose load [87], high adiponectin serum levels [88], the absence of phosphatase and tensin homolog deleted from chromosome 10 (PTEN) [89], connexin 43 inhibition [90], calcium release-activated calcium channel protein 1 (Orai1) deficiency [91], transforming growth factor-β1 (TGF-β1) [92], and extralarge Gα subunit (XLas) [93]. Some of the drugs that block its synthesis are PKC inhibitors [59], FGFR-specific inhibitor SU5402 [35], molidustat [63], holo-transferrin [63], insulin [89,94], tocotrienol [95], sevelamer [96], vitamin A [97], and pamidronate [98].

A gradual increase in the expression of FGF23 levels during osteocyte maturation has been described at both mRNA and protein levels. On the contrary, the levels of FGF23 mRNA in osteoblasts progressively decrease [99,100]. Given the co-expression of FGF23 with the early osteocyte marker E11/gp38, a potential role of FGF23 as a marker of early osteocyte maturation has been suggested [101]. A differential expression of FGF23 according to age has also been described, being higher in the bone of adult individuals with respect to younger ones [7,99,102]. Overall, FGF23 expression is dynamically regulated by systemic factors and intrinsic bone-derived factors across diverse physiological and pathological contexts (Figure 1).

## 4 Paracrine actions of FGF23

### 4.1 Paracrine FGF23 signalling in bone cells and remodelling

**Figure 2:**
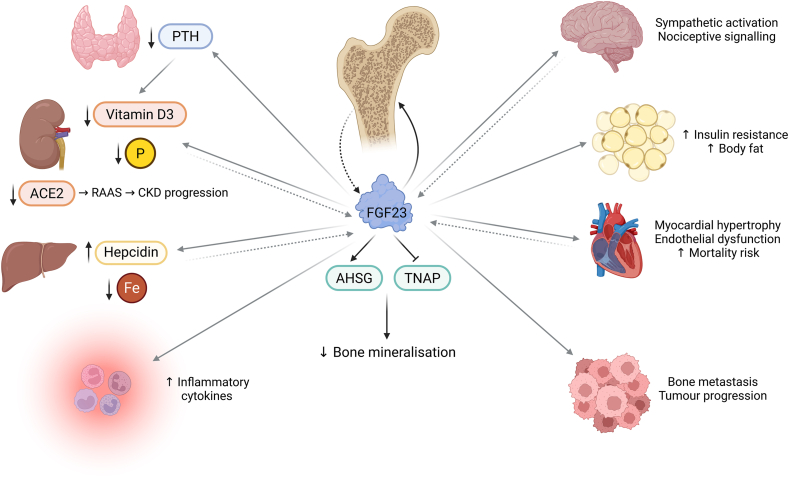
**Paracrine and endocrine actions of FGF23.** FGF23 is predominantly synthesised by osteocytes and osteoblasts in bone (dashed black arrow), where it exerts paracrine actions within the bone microenvironment (solid black arrow). Locally, FGF23 reduces bone mineralisation through induction of fetuin-A (AHSG) and inhibition of tissue-nonspecific alkaline phosphatase (TNAP). FGF23 also exerts broad endocrine actions on mineral metabolism, cardiovascular function, inflammation and energy homeostasis (solid grey arrows). In the parathyroid gland, FGF23 suppresses parathyroid hormone (PTH) secretion. In the kidney, FGF23 promotes phosphaturia, suppresses active vitamin D3 synthesis, and downregulates angiotensin-converting enzyme 2 (ACE2), contributing to renin–angiotensin–aldosterone system (RAAS) activation and chronic kidney disease (CKD) progression. In the liver, FGF23 modulates iron metabolism by increasing hepcidin expression and reducing circulating iron levels. Within the immune system, FGF23 enhances inflammatory cytokine production. In the nervous system, FGF23 facilitates sympathetic activation and nociceptive signalling. In adipose tissue, elevated FGF23 is associated with increased insulin resistance and body fat accumulation. Cardiovascular effects include myocardial hypertrophy, endothelial dysfunction and increased mortality risk. In cancer, FGF23 has been implicated in tumour progression and bone metastasis. Although bone is the primary source of this phosphatonin, lower levels of FGF23 expression have also been detected in other tissues, including kidney, liver, heart and brain (dashed grey arrows).

FGF23 exerts paracrine actions on bone mineralisation through fetuin-A (AHSG) and tissue-nonspecific alkaline phosphatase (TNAP) (Figure 2). FGF23 is an activator of AHSG, a protein synthesized in the liver which has anticalcifying properties with relevance at the bone matrix level and in ectopic calcifications. Mattinzoli et al. described how the increase in FGF23 in the preosteoblast cell line MC3T3-E1 was associated with an increase in AHSG, confirming that AHSG can be synthesized at the bone level. In addition, coinciding with the increase in FGF23 and AHSG, an elongation of the dendritic processes of the osteocytes was observed [100]. In subsequent differentiation experiments with mesenchymal stromal cells (MSCs), a progressively higher expression of FGF23, without an increase in its secretion, was observed. The behaviour of FGF23 and AHSG was similar to that previously described, further confirming that high doses of FGF23 in MC3T3-E1 cells stimulated proliferation and inhibited mineralisation of MSCs [103].

Besides, FGF23 directly inhibits TNAP expression in osteoblasts at the mRNA and protein levels, α-KL-independently, through its binding to FGFR3 and consequent activation of extracellular signal-regulated kinase (ERK) pathway. The suppression of TNAP leads to decreased degradation of pyrophosphate, which accumulates in the extracellular matrix, and indirectly inhibits osteopontin, impairing bone mineralisation [25,104]. Due to these effects, FGF23 overexpression has been inversely correlated with osteoid thickness [76]. Numerous bone diseases have been related to an increase in FGF23 transcription, including hypophosphatemic rickets [105,106], fibrous dysplasia [107,108], and tumour-induced osteomalacia [109]. Even though FGF23's primary driver of osteomalacia is its endocrine action on the kidney, increased bone expression of FGF23 may also contribute to altered bone mineralisation and osteomalacia due to TNAP suppression [25,110].

### 4.2 Paracrine FGF23 signalling in CKD-associated bone disease

Bone FGF23 expression has also been described to correlate positively with CKD stages [111,112]. In a study of bone histomorphometry in patients with CKD stages 2–5, FGF23 and DMP1 expression was upregulated in trabecular bone and inversely related to osteoid accumulation. This increase in DMP1 is interpreted as an attempt to counteract the increase in FGF23 secondary to progressive phosphate overload during CKD [113]. In another study in adult patients on dialysis (CKD stage 5), high bone turnover disease was associated to higher values of serum FGF23 [111,112]. In renal osteodystrophy, which occurs as a result of CKD, FGF23 marks osteocytes in the early stages of maturation, whose number increases while their apoptosis decreases. Primary osteoblasts extracted from patients preserved their altered mineralisation capacity *in vitro*, although this was not modified by the addition of FGF23 [101]. The Wnt pathway, involved in osteoblastic differentiation, was suppressed by an increment of the Dickkopf1 inhibitor mediated by high levels of FGF23 [114,115].

On the other hand, *Fgf23*^−/−^ mice have shown delayed bone growth and abnormal development with altered morphology, wider epiphyses, and a significant reduction in mineral content associated with increased bone osteoid. Likewise, hypertrophic chondrocytes are absent from the structure of articular cartilage [10]. *Fgf23*^−/−^ mice also presented an altered dentoalveolar complex, with disruption of odontoblast layer and ectopic matrix deposition in pulp chambers, mineralisation defects and an increase of apoptotic cells in the mandible compared to wild type [116]. In regard to this, a deficiency of FGF23 has been documented in patients with hip fracture [117] or familial hyperphosphatemic tumoral calcinosis [118].

Similarly, reduced levels of FGF23 and several of its receptors have been found in a rat model in which osteoporosis was established by ovariectomy [119]. However, FGF23 trabecular expression did not correlate with any parameters of skeletal mineralisation nor with bone DMP1 and sclerostin expression in a cohort of patients with monogenic forms of osteoporosis [120], which contrasts with the observations made in patients with renal osteodystrophy [113] and *Hyp* mice [92]. Together, these findings support a direct paracrine role of FGF23 in regulating bone mineralisation and osteocyte function under both physiological and pathological conditions.

## 5 Endocrine actions of FGF23

### 5.1 Phosphocalcium homeostasis and vitamin D regulation

FGF23 is the main regulator of phosphate at the systemic level. FGF23 acts at the renal level by inhibiting the type II sodium-phosphate cotransporters (NaPi-2a and NaPi-2c) in the proximal tubule, both reducing their expression [121] and promoting their internalization [122], thereby reducing phosphate reabsorption [123]. FGF23 also globally decreases the synthesis of active vitamin D3 by inhibiting renal 1α-hydroxylase and activating 24α-hydroxylase [7,25,123,124], thereby limiting intestinal absorption of calcium and phosphate and contributing to a negative net phosphate balance. In addition, FGF23 inhibits PTH transcription and secretion by activating the MAPK pathway through ERK1/2 phosphorylation and increasing early growth response 1 (EGR-1) mRNA levels [125]. This inhibition of PTH, among other effects, leads to reduced release of calcium and phosphate from bone, lower tubular reabsorption of calcium, increased phosphate excretion at the renal level, and further suppression of active vitamin D3 synthesis (Figure 2) [125,126]. Consistent with this, a decrease in FGF23 has been described in situations of diet-induced hypocalcaemia, despite high levels of PTH and active vitamin D3, possibly to avoid a suppressive effect of FGF23 on vitamin D activation that would worsen hypocalcaemia [67]. These endocrine actions position FGF23 as a central regulator of systemic phosphate and vitamin D metabolism. Notably, FGF23's effects on phosphate metabolism and vitamin D synthesis are key contributors to the development of osteomalacia, as low phosphate levels and reduced vitamin D3 synthesis impair hydroxyapatite formation, leading to altered bone mineralisation [84] (Table 1).

**Table 1:** Paracrine and endocrine actions of FGF23 across tissues and organ systems.

Target tissue	Receptor context	Main signalling pathway	Biological effect	Pathological relevance	References
Bone	FGFR3 (α-KL-independent)	ERK	↓ TNAP, ↑ PPi	Osteomalacia	[25,104,110]
Kidney	FGFR1/α-KL	NaPi-2a andNaPi-2c, Vit D enzymes	Phosphaturia, ↓ ACE2	CKD, CV risk, osteomalacia	[7,8,25,84,121,[123], [124], [125], [126],^171^]
Parathyroid	FGFR1/α-KL	ERK–EGR1	↓ PTH	Mineral imbalance	[125,126]
Heart	FGFR4 (α-KL-independent)	Calcineurin–NFAT	Hypertrophy	Heart failure	[26,153,154,156,157,160]
Immune cells	FGFR2	PKA/Rasp1	↓ Neutrophil migration	Immune dysfunction	[170]
Cancer/bone niche	FGFR(s) unidentified	ERK–EGR1	Angiogenesis, tumour progression	Metastasis	[[146], [147], [148], [149]]
Nervous system	FGFR1	Neuronal depolarisation	Sympathetic activation	CV risk	[178,179]

ACE2, angiotensin-converting enzyme 2; α-KL, α-Klotho; CKD, chronic kidney disease; CV, cardiovascular; EGR1, early growth response 1; ERK, extracellular signal-regulated kinase; FGFR, fibroblast growth factor receptor; NFAT, nuclear factor of activated T cells; NaPi-2a, type IIa sodium–phosphate cotransporter; PKA, protein kinase A; PPi, pyrophosphate; PTH, parathyroid hormone; TNAP, tissue-nonspecific alkaline phosphatase; Vit D, active vitamin D3.

### 5.2 Energy metabolism and insulin sensitivity

Shimada et al. first described the presence of hypoglycaemia in *Fgf23*^−/−^ mice [10]. Subsequently, Hesse et al. provided evidence for a role of FGF23 and active vitamin D3 in carbohydrate metabolism. In an experiment with *Fgf23*^*−/−*^ mice, vitamin D receptor (VDR) mutant mice (with a non-functioning VDR), and double mutant mice (*Fgf23*^*−/−*^ and *Vdr*^*−/−*^) showed that, while the first group developed hypoglycaemia and increased peripheral insulin sensitivity, these alterations did not exist in animals lacking vitamin D signalling [127]. FGF23 has been associated with type 2 diabetes mellitus (T2DM) [128], insulin resistance in patients with CKD stages 3–5 [[129], [130], [131]], obese patients with prediabetes [132], diabetic patients without insulin treatment [130], and diabetics with non-alcoholic fatty liver disease (NAFLD) [133]. Resistin, an adipokine that increases in situations of resistance to this hormone, has also been positively associated with FGF23 in patients with CKD [131] and T2DM [134].

Regarding body adiposity, *Fgf23*^*−/−*^ mice had a reduction in total body fat mass compared to wild type, *Vdr*^*−/−*^ mice, and doble mutant mice [135]. In humans, high levels of FGF23 have been associated with increased body mass index (BMI), waist circumference (WC), and visceral fat area [136], as well as dyslipidaemia [137]. In a cross-sectional study of more than 1,000 individuals, the phosphatonin was positively associated with BMI and WC in the population without CKD, but not in those with this condition [131]. Together, these findings suggest a functional link between FGF23, vitamin D signalling and systemic energy metabolism, whereby FGF23 deficiency is associated with hypoglycaemia and increased peripheral sensitivity to insulin, while elevated FGF23 levels are linked to increased tissue insulin resistance and greater total and visceral body fat, although the underlying mechanisms remain incompletely defined.

### 5.3 Inflammation and iron homeostasis

FGF23 has been associated with inflammatory diseases such as liver cirrhosis [138], sepsis [51], and several rheumatic diseases, including rheumatoid arthritis [139], systemic lupus erythematosus [140,141], and axial spondyloarthritis [142]. Moreover, in a cross-sectional study of 1,040 individuals, this phosphatonin was positively associated with circulating levels of interleukin-6, interleukin-10, and C-reactive protein [131]. In patients with sepsis, increased levels of pro-inflammatory cytokines (TNF, IL-1β) and LPS can cause transient hypophosphatemia mediated by induction of FGF23 [8,51]. Beyond this associations, FGF23 has been shown to increase the number of resident macrophages and to stimulate their secretion of TNF-α [54], as well as to promote hepatic production of inflammatory cytokines in an α-KL independent manner [27].

Inflammatory regulation of FGF23 also has important consequences for iron metabolism and anaemia. Increased production of cFGF23 has been described in association with elevated EPO expression, increased hepcidin expression, and decreased serum iron levels (Figure 2) [52,55,143]. This sequestration of serum iron, resulting from the activation of cells of the mononuclear phagocytic system via toll-like receptor 4, was prevented by blockade of FGF23 signalling [55]. In contrast, increased circulating cFGF23 has been linked to reduced hepatic hepcidin synthesis by antagonizing bone morphogenetic protein (BMP)2- and BMP9-dependent stimulation [144]. This effect has been proposed as an adaptive regulatory mechanism to increase iron bioavailability and avoid hypoferremia during acute inflammation. In line with this, increased EPO levels have been shown to directly stimulate FGF23 production in both bone and bone marrow, contributing to changes in iron homeostasis and the development of anaemia [145]. Overall, these observations are compatible with the involvement of FGF23 in inflammatory signalling networks and in the modulation of iron homeostasis during inflammatory states, with context-dependent adaptive and pathological effects.

### 5.4 Cancer progression and tumour-bone interactions

FGF23 has been implicated in the biology of both benign and malignant neoplasms. Human breast cancer cells have been described to lack FGF23 expression *in vitro* or after injection into mouse lymph nodes, whereas they express FGF23 strongly upon metastatic colonisation of bone [146]. FGF23 expression has been found in prostate tumour tissue as well, possibly acting as a local growth factor, favouring its growth and invasion capacity [147]. In a human *ex vivo* 3D bone model, osteocytes co-cultured with metastatic prostate cancer cells also increased FGF23 expression [148]. Similarly, co-culture of multiple myeloma (MM) cells induces a pro-angiogenic response in MLOA5 murine osteocytes through transcription of FGF23 and EGR-1 [149] (Table 1), and signalling downstream of these factors induces heparanase expression, favouring osteolytic lesions [150]. Low FGF23 serum levels have been associated with increased survival and with a lower incidence rate of bone-related events, such as pathological fractures or metastases [151]. In addition, three SNPs in FGF23 have been associated with an increased risk of prostate cancer in the Korean population [152]. Collectively, the available evidence supports a role for FGF23 in tumour-bone interactions, particularly in the context of bone metastasis and osteolytic disease, where local and systemic FGF23 signalling may influence tumour progression, angiogenesis and skeletal complications, although the underlying mechanisms and clinical implications remain incompletely understood.

### 5.5 Cardiovascular hypertrophy and dysfunction

High circulating levels of FGF23 have been associated with heart failure. Exposure of cardiomyocytes to FGF23 increases intracellular calcium levels and induces myocardial hypertrophy through FGFR-dependent activation of the calcineurin/nuclear factor of activated T cells (NFAT) signalling pathway [153,154]. Consistently, Grabner et al. prevented the development of myocardial hypertrophy in rats with CKD using an FGFR4-blocking antibody and inhibition of the PLCγ/calcineurin/NFAT pathway in the absence of α-KL as co-receptor [26] (Table 1). In addition, treatment of mouse ventricular tissue with FGF23 increased the expression of cardiac hypertrophy markers such as atrial natriuretic peptide (ANP) and brain natriuretic peptide (BNP) and enhanced muscle contractility *ex vivo*. However, hearts from type IV collagen alpha 3 chain (*Col4a3)*^*−/−*^ mice, an animal model of CKD, exhibited impaired left ventricular function with reduced fractional shortening and ejection fraction without overt cardiac hypertrophy [155].

An study in humans showed that FGF23 levels were higher in obese adolescents than in normal-weight individuals, and were further increased in those with concentric or eccentric myocardial hypertrophy [156]. Cardiac conduction abnormalities such as ventricular extrasystoles, ventricular tachycardia bursts, and QT interval prolongation on electrocardiogram (features associated with sudden cardiac death) have also been reported in association with elevated FGF23 [157]. At the vascular level, FGF23 has been linked to endothelial dysfunction, with reduced nitric oxide production and impaired acetylcholine-induced vasorelaxation [158], as well as to calcium deposition within coronary atherosclerotic plaques and progressive renal dysfunction [159]. Clinically, elevated FGF23 levels have been associated with increased mortality in patients with acute myocardial infarction complicated by cardiogenic shock and renal impairment [160], as well in critically ill populations [161]. Experimental and clinical evidence therefore indicates that elevated FGF23 contributes to adverse cardiovascular remodelling, vascular dysfunction and increased mortality risk, particularly in the context of metabolic and renal disease.

### 5.6 Chronic kidney disease

Several kidney disorders have been associated with increased FGF23 levels, including metabolic acidosis [162,163], acute renal failure [[164], [165], [166]], chronic uraemia [12], and CKD [8]. Moreover, elevated plasma FGF23 has been linked to increased urinary phosphate excretion and declining renal function parameters both in young healthy individuals [167] and in patients with CKD [168]. During CDK progression, multiple factors converge to drive a progressive rise in FGF23, including anaemia, systemic inflammation, and disturbances in phosphorus-calcium metabolism involving PTH, phosphate, and active vitamin D3. Notably, increased serum FGF23 can be detected early in this process [8,169]. Functionally, FGF23 impairs immune responses in CKD by inhibiting polymorphonuclear neutrophil adhesion and transendothelial migration through binding with FGFR2, activation of protein kinase A (PKA), and modulation of the small GTPase Rasp1 [170]. In addition, FGF23 promotes the transcription of genes related to fibrosis and inflammation, including lipocalin-2 (LCN2), TNF-α and monocyte chemoattractant protein-1 (MCP-1), and activates signalling pathways associated with TGF-β, LCN2, and TNF-α [23,24].

FGF23 signalling has also been shown to suppress angiotensin-converting enzyme 2 (ACE2), a negative regulator of the renin-angiotensin-aldosterone system (RAAS) that degrades angiotensin I and II (Figure 2). Dysregulation of this axis affects blood pressure control, extracellular volume and plasma concentration of Na^+^ and K^+^, with important implications for cardiovascular disease and mortality [171] (Table 1). Consistent with this, administration of recombinant FGF23 has been reported to antagonise the protective effects of RAAS-blocking therapies on CKD progression [24]. Conversely, Clinkenbeard et al. have proposed the rising of FGF23 in CKD as an initial adaptive response aimed at limiting vascular complications (such as aortic calcification and cardiac hypertrophy) related to hyperphosphataemia and secondary hyperparathyroidism, although sustained elevation is ultimately associated with increased mortality [172]. Overall, current evidence indicates that FGF23 plays a multifaceted role in CKD pathophysiology, exerting both adaptive and maladaptive effects on immune function, cardiovascular regulation and disease progression.

### 5.7 Neural and autonomic regulation

FGF23 has been associated with disorders of the nervous system, such as neurofibromatosis [173], memory and learning deficits, and hippocampal-dependent cognitive impairment [174,175], as well as axonal loss in the frontal lobe in the context of CKD [176]. In addition, cerebrospinal fluid concentration of FGF23 has been linked to impulsive behaviour [177]. Functionally, FGF23 facilitates nociceptive pain transmission through signalling of peripheral sensory neurons [178], and promotes the activation of the sympathetic nervous system by inducing depolarisation of presympathetic neurons in the rostral ventrolateral medulla [179]. These bulbospinal neurons project connect with the preganglionic sympathetic neurons of the spinal cord, thereby contributing to sympathetic activation, with systemic consequences, including effects on blood pressure regulation. In both nociceptive and autonomic pathways, FGF23-induced neuronal depolarisation is mediated through FGFR1 [178,179] (Table 1). These observations suggest that FGF23 influences neural function, with potential implications for cognitive, behavioural and cardiovascular regulation.

## 6 Key knowledge gaps and future research

FGF23 has emerged as a multifaceted bone-derived hormone with broad implications in mineral homeostasis, systemic metabolism, and inflammation. While its role in bone mineralisation and phosphate metabolism is well-established, its broader actions in regulating energy homeostasis, inflammation, and cardiovascular health remain less understood and warrant further investigation.

A critical area for further exploration is FGF23's involvement in insulin resistance and energy metabolism. As FGF23 levels increase in metabolic disorders like obesity and T2DM, correlating with insulin resistance, its role as modulator of insulin sensitivity and energy homeostasis becomes more apparent. Understanding the molecular mechanisms underlying FGF23's interactions with glucose metabolism pathways and adipose tissue signalling pathways could provide novel insights into its role in insulin resistance and its potential as a therapeutic target for metabolic diseases.

Another key aspect that requires further investigation is FGF23's role in bone and mineral dysregulation, particularly in diseases such as chronic kidney disease (CKD) and osteomalacia. Although FGF23's suppression of TNAP and modulation of osteocyte function are well-documented, the interplay between paracrine FGF23 effects in bone and systemic metabolic disturbances remains largely unexplored. Future research should elucidate whether these paracrine effects contribute to pathophysiology in CKD, cardiovascular disease, and osteoporosis.

Furthermore, FGF23's involvement in inflammatory pathways and iron homeostasis presents an important research avenue. Elevated levels of FGF23 are associated with immune dysregulation in chronic diseases, yet its dual protective and pathological role needs to be clarified. Investigating how FGF23 interacts with inflammatory cytokines in diseases like autoimmunity and CKD could provide essential insights into its role in regulating iron metabolism and the immune response, as well as its potential for therapeutic modulation.

Another key area warranting further investigation is the role of FGF23 in cardiovascular disease, where this bone-derived hormone promotes cardiovascular hypertrophy and dysfunction and increases mortality risk. Further research is needed to identify how FGF23 signalling pathways contribute to cardiovascular disease progression, and whether targeting FGF23 could offer new therapeutic strategies for metabolic and cardiovascular diseases without disrupting bone mineralisation and phosphate control.

In conclusion, FGF23 has emerged as a bone-derived hormone with pleiotropic actions that extend well beyond its canonical role in phosphate and vitamin D homeostasis. Through tightly regulated synthesis, post-translational processing and context-dependent receptor interactions, FGF23 integrates paracrine control of bone mineralisation with endocrine regulation of metabolic, inflammatory, cardiovascular, and renal pathways (Figure 2). A deeper understanding of the mechanisms governing FGF23 production, processing and tissue-specific signalling will provide new insights into disease mechanisms and open avenues for new therapeutic strategies aimed at selectively modulating FGF23 pathways without disrupting mineral homeostasis.

## Funding information

The authors disclose receipt of the following financial support for the research and publication of this article: Instituto de Salud Carlos III (ISCIII) through projects with grant number PI22/00407, PI25/01319, CPII20/00026, and CD24/00029, co-funded by the European Union through “Fondo de Investigación Sanitaria” from Fondo Eu-ropeo de Desarrollo Regional (FEDER)/Fondo Social Europeo (FSE); Xunta de Galicia (grant number IN607B-2025/15); the Mutua Madrileña Foundation (grant number MMA 2025); and Instituto de Investigación Sanitaria de Santiago de Compostela (IDIS). Support was also associated with personnel, as follows: M.P-R. [CD24/00029], A.P-P. [ENDOTARGET, Grant agreement ID: 101095084], M.C.-S. [ENDOTARGET, Grant agreement ID: 101095084], L.Z.-V. [IDIS 2025], A.A.-P. [IDIS 2022], and R.G. [ISCIII and SERGAS through the Miguel Servet II program CPII20/00026].

## CRediT authorship contribution statement

**Antía Crespo-Golmar:** Writing – original draft, Investigation. **María Piñeiro-Ramil:** Writing – original draft, Supervision, Investigation. **Andrés Pazos-Pérez:** Validation, Supervision, Investigation. **María Crugeiras-Sampedro:** Visualization, Investigation. **Lorena Zas-Veiga:** Visualization, Investigation. **Djedjiga Ait Eldjoudi:** Writing – review & editing, Investigation. **Ana Suárez-Rodríguez:** Writing – original draft, Investigation. **Alberto Jorge-Mora:** Writing – review & editing. **Ana Alonso-Pérez:** Writing – review & editing, Supervision, Conceptualization. **Rodolfo Gómez:** Writing – review & editing, Project administration, Conceptualization.

## Declaration of competing interest

The authors declare that they have no known competing financial interests or personal relationships that could have appeared to influence the work reported in this paper.

## Data Availability

No data was used for the research described in the article.
